# Primary care providers’ perspectives on barriers and enablers influencing preconception care provision for African migrant and refugee women: qualitative interviews

**DOI:** 10.1093/heapro/daag091

**Published:** 2026-07-17

**Authors:** Beyene Meressa Adhena, Anna Chapman, Renee Fiolet, Alison M Hutchinson

**Affiliations:** School of Nursing and Midwifery, Deakin University, 1 Gheringhap Street, Geelong, Victoria 3220, Australia; Deakin Centre for Quality and Patient Safety Research, Deakin Institute for Health Transformation, Deakin University, 1 Gheringhap Street, Geelong, Victoria 3220, Australia; Deakin Centre for Quality and Patient Safety Research, Deakin Institute for Health Transformation, Deakin University, 1 Gheringhap Street, Geelong, Victoria 3220, Australia; Office of the Executive Dean of Health, Faculty of Health, Deakin University, 1 Gheringhap Street, Geelong, Victoria 3220, Australia; School of Nursing and Midwifery, Deakin University, 1 Gheringhap Street, Geelong, Victoria 3220, Australia; Deakin Centre for Quality and Patient Safety Research, Deakin Institute for Health Transformation, Deakin University, 1 Gheringhap Street, Geelong, Victoria 3220, Australia; School of Nursing and Midwifery, Deakin University, 1 Gheringhap Street, Geelong, Victoria 3220, Australia; Deakin Centre for Quality and Patient Safety Research, Deakin Institute for Health Transformation, Deakin University, 1 Gheringhap Street, Geelong, Victoria 3220, Australia; Barwon Health, 322 Ryrie Street, Geelong, Victoria 3220, Australia

**Keywords:** preconception care, primary health care, migrants and refugees, African women, health disparities, implementation science

## Abstract

Preconception care (PCC) is an important preventive strategy for improving maternal and child health, yet its routine delivery in primary care remains inconsistent, particularly for populations experiencing inequities. We explored primary care providers’ perspectives on barriers to and enablers of providing PCC to African migrant and refugee women in Victoria, Australia. Eleven primary care providers (four general practitioners, four practice or refugee health nurses, and three maternal and child health nurses) participated in semi-structured interviews. Interviews were transcribed verbatim, de-identified, and analysed using a hybrid deductive-inductive thematic approach guided by the Theoretical Domains Framework, with subthemes mapped to the Capability, Opportunity, Motivation-Behaviour (COM-B) domains. Providers described PCC as important, yet acknowledged that its delivery was inconsistent and often embedded within other consultations rather than delivered in a standalone consultation. Enablers included preventive intent, clinical and communication skills, teamwork, continuity of care, trusted relationships, and the staged use of routine visits to introduce PCC over time. Barriers included limited time, funding and clinic resources, gaps in PCC training, inconsistent interpreter access, and broader social and structural factors influencing women’s engagement with care. Improving equitable PCC delivery for African migrant and refugee women will require strategies that strengthen provider capability and address organizational, relational, and system-level conditions that support routine, culturally responsive PCC in primary care.

Contribution to Health Promotion

- Primary care providers recognized that preconception care (PCC) can support healthier pregnancies, but acknowledged that it is not delivered consistently to African migrant and refugee women in Victoria.
- Providers identified barriers to preconception care, including limited time, funding and clinic resources, gaps in PCC training, and inconsistent access to interpreters.
- Trusted relationships, teamwork, and using routine visits to raise preconception care over time were seen as important enablers.
- Health promotion in primary care could be strengthened through clearer care pathways, better language support, and clinic systems that support equitable care.

## Background

Preconception care (PCC) is defined as a package of biomedical, behavioural, and social health interventions delivered before pregnancy to improve maternal and infant outcomes and reduce avoidable inequities across the life course (World Health Organization [WHO] 2013). Global guidance conceptualizes PCC as an upstream prevention strategy that extends beyond discrete clinical checks to include health literacy, chronic disease management, nutrition, psychosocial wellbeing, and attention to social determinants of health when embedded within primary care systems (WHO 2013, Shawe *et al*. 2020). Preconception care is increasingly framed as a universal and opportunistic approach, recognizing that pregnancy intention is fluid and often unarticulated (Hill *et al*. 2020, Jacob *et al*. 2020).

Since 2013, international recommendations have increasingly supported the integration of PCC into routine care across the reproductive life course, including adolescence, young adulthood, and the interpregnancy period, using approaches that do not rely on explicit pregnancy planning (WHO 2013, Hill *et al*. 2020, Jacob *et al*. 2020). In Australia, the current clinical guideline specifies core PCC components, including folate and iodine supplementation, optimization of chronic conditions, immunization, medication review, screening for sexually transmitted infections, alcohol and tobacco use, attention to nutrition and weight, and attention to mental health, alongside an explicit equity focus for culturally and linguistically diverse populations (Royal Australian College of General Practitioners [RACGP] 2025). In Australia, as the main community-based entry point to the health system, primary care is well suited to the delivery of preventive health promotion and early intervention. Primary care is, therefore, positioned as a key platform for PCC delivery due to its broad population reach, continuity of care, and repeated opportunities to provide preventive education and opportunistic reproductive health advice before pregnancy (Australian Institute of Health and Welfare [AIHW] 2016, Atrash and Jack 2020).

Despite WHO guidance supporting the integration of PCC within the continuum of care, it remains inconsistently delivered in primary care (WHO 2013, Withanage *et al*. 2023, Caut *et al*. 2025). In Australia and other high-income settings, PCC discussions are often selective and dependent on individual clinician initiative rather than embedded within routine and systematic workflows (Griffiths *et al*. 2020, Caut *et al*. 2025). Commonly cited barriers include limited consultation time, competing priorities, and insufficient structural supports (Mengesha *et al*. 2017, Caut *et al*. 2025). Audit studies of primary care records further show that documentation of preconception risk factors is often incomplete and inconsistently acted on across care episodes (Withanage *et al*. 2024, 2025). However, less is known about how PCC is understood, prioritized, and experienced among culturally diverse women, including African migrant and refugee women (Withanage *et al*. 2024, 2025).

Equitable access to PCC is a particular concern for migrant and refugee women, whose engagement with preventive and reproductive health care is shaped by migration-related disadvantages, including language barriers, limited familiarity with health systems and entitlements, financial and transport constraints, and experiences of racism or discrimination, rather than individual choice alone (Mengesha *et al*. 2017, Victorian Government 2018, Lebano *et al*. 2020, Khatri and Assefa 2022). Conceptual models of healthcare access further highlight the structural nature of these inequities, emphasizing that access is multi-dimensional and encompasses approachability, acceptability, availability, affordability, and appropriateness (Levesque *et al*. 2013, Rogers *et al*. 2020). For culturally and linguistically diverse communities, these dimensions may be compromised by language barriers, limited health literacy, difficulties navigating services, and a lack of culturally responsive care (Rogers *et al*. 2020, Raymundo *et al*. 2021). Australian evidence similarly shows that inequitable healthcare access for culturally and linguistically diverse communities is shaped by systemic barriers related to communication, cultural responsiveness, and navigation of the health system (Federation of Ethnic Communities’ Councils of Australia [FECCA] 2022, Khatri and Assefa 2022, Victorian Government 2018).

In Victoria, African migrant and refugee communities face intersecting challenges related to discrimination, trauma, settlement stressors, and mismatches between service design and community needs (Victorian Government 2018). Population-level analyses demonstrate poorer perinatal outcomes among East African-born women in Victoria, including higher risks of perinatal mortality, small-for-gestational-age birth, and very preterm birth (Belihu *et al*. 2016). Evidence from other high-income contexts also shows inequities in key preconception indicators, including low folic acid uptake among some migrant groups (Nilsen *et al.* 2019). Qualitative studies indicate that limited awareness of PCC, unplanned pregnancy, competing settlement priorities, and preferences for culturally safe information shape women’s engagement with preventive care (Mazza and Chapman 2010, Rogers *et al*. 2020, Kasper *et al*. 2022). Together, this literature suggests that reliance on pregnancy planning or women’s initiation of care may shape who accesses PCC (Griffiths *et al*. 2020, Hill *et al*. 2020). Efforts to improve equitable access to PCC must avoid reinforcing medicalized or stigmatizing approaches to women’s reproductive lives, particularly where cultural safety, autonomy, and choice are not foregrounded (Pentecost and Meloni 2020, Waggoner and Pentecost 2025). From rights-based and reproductive justice perspectives, PCC is most appropriate when it is voluntary, non-coercive, and responsive to women’s priorities (Mckercher 2020, Azugbene 2023).

From a health promotion perspective, equity-oriented PCC requires attention not only to women’s knowledge and preferences, but also to organizational, behavioural, and policy determinants that shape PCC provision within primary care as part of upstream prevention and equitable, culturally responsive service delivery (Boyle *et al*. 2022, Doherty *et al*. 2022). This aligns with the Ottawa Charter’s emphasis on reorienting health services and creating supportive environments (WHO 1986), including systems-level change and community engagement (Lunnay *et al*. 2025). It also aligns with evidence on the importance of trust, culturally sensitive care, native-language information, and organizational support for migrant women’s health literacy and access (Jubran Leksell *et al*. 2025). To understand these influences, conceptual frameworks are needed to explain provider behaviour and the factors shaping PCC provision in complex primary care contexts. The Theoretical Domains Framework (TDF) offers a structured way to identify determinants of healthcare professional behaviour and clinical practice (Cane *et al*. 2012). The Capability Opportunity Motivation-Behaviour (COM-B) model conceptualizes behaviour as a function of capability, opportunity, and motivation (Michie *et al*. 2011).

Within primary care, providers play a central role in shaping whether PCC is offered opportunistically, how it is framed, and whose needs are prioritized within constrained consultations (AIHW 2016, RACGP 2025). However, equitable PCC provision for migrant and refugee women depends not only on individual providers, but also on the organizational and system-level conditions that enable or constrain delivery (Furneaux *et al*. 2016, Mengesha *et al*. 2017). Although task-sharing, multidisciplinary models, and whole-of-practice approaches have been proposed to strengthen PCC delivery, there is limited context-specific evidence on how primary care providers understand the barriers and enablers to providing PCC for African migrant and refugee women in Victoria (Furneaux *et al*. 2016, Walker *et al*. 2020).

As PCC provision is a provider behaviour shaped by individual, organizational and system-level conditions, the TDF informed the identification of potential determinants of PCC provision and the development of interview questions exploring providers’ knowledge, skills, professional roles, beliefs, social influences, and environmental constraints. The COM-B was then used to organize these determinants according to capability, opportunity, and motivation and to support interpretation of how they shaped PCC provision. Within a health promotion framing, these frameworks helped identify whether change was needed at the provider, service or system level. Accordingly, the study addressed the following research question: What are the barriers to and enablers of the provision of PCC to African migrant and refugee women living in Victoria, as perceived by primary care providers?

## Methods

### Study design

We selected a qualitative approach to enable detailed exploration of providers’ experiences, practices, and perceptions within real-world primary care settings, where PCC delivery is shaped by organizational, behavioural, and system-level factors. We used a qualitative exploratory descriptive design (Hunter *et al*. 2019), informed by the principles of qualitative description and general qualitative inquiry (Sandelowski 2010). This approach is well-suited to applied health research, where the aim is to generate a contextually grounded understanding and practice-relevant insights into participants’ experiences and perspectives. To support interpretation of behavioural influences on PCC provision, the study was informed by the TDF and COM-B model. The TDF is an integrative behavioural framework that synthesizes key constructs from multiple behaviour change theories into 14 domains: knowledge; skills; social/professional role and identity; beliefs about capabilities; optimism; beliefs about consequences; reinforcement; intentions; goals; memory, attention and decision processes; environmental context and resources; social influences; emotion; and behavioural regulation (Cane *et al*. 2012). The COM-B model proposes that behaviour is part of an interacting system involving capability, opportunity, and motivation (Michie *et al*. 2011). In this study, findings relating to the TDF domains were mapped onto the COM-B model components of capability, opportunity, and motivation to support interpretation of influences on PCC provision. Hence, the TDF supported systematic identification of determinants, while the COM-B model provided an overarching interpretive structure for understanding providers’ practices.

### Setting

The study was conducted in general practice and maternal and child health services, key primary care settings for the opportunistic delivery of PCC in Victoria, Australia (RACGP 2025).

### Participants and recruitment

Eligible participants were general practitioners (GPs), practice nurses, refugee health nurses, and maternal and child health (MCH) nurses who had a minimum of 1 year of experience working in primary care settings serving African migrant and refugee populations in Victoria. The 1-year minimum was used to ensure that participants had sufficient experience of routine practice to reflect on barriers and enablers to PCC provision in these settings.

Purposive sampling was used to recruit eligible primary care providers. Recruitment commenced through general practices, refugee health services, and council-run maternal and child health centres. On behalf of the researchers, the clinic reception staff handed the Plain Language Statement and Consent Form to providers, and interested providers contacted the doctoral candidate directly by phone and/or email. Snowball sampling was subsequently used, whereby participating providers shared study information with colleagues to support recruitment of additional participants.

Participant recruitment continued until the data were judged to hold sufficient information power to address the study aim, considering the specificity of the sample, the richness of participants’ accounts, and the theoretically informed analysis (Malterud *et al*. 2021). The research team determined that this had been achieved after nine interviews; two further interviews did not introduce new barriers or enablers, alter analytic categories, or change the COM-B interpretation. Recruitment concluded with 11 providers: four GPs, four practice or refugee health nurses, and three MCH nurses.

### Data collection

Data were collected through individual semi-structured interviews conducted between August 2024 and March 2025. Interviews were conducted by the first author, an international PhD candidate from Ethiopia with training and prior experience in qualitative research methods. This background helped the interviewer recognize issues raised by participants about migration, language, settlement, and cultural expectations in reproductive health care. We also considered how differences between the interviewer and participants could shape questioning about PCC, rapport during interviews, and interpretation of provider accounts about culture, migration, and service access for African migrant and refugee women. Debriefing and analytic discussions among the research team were used to examine how background and prior knowledge of PCC may have shaped data collection and interpretation. No prior relationship existed between the interviewer and participants. Participants were informed of the researcher’s background and the study purpose before providing consent. Interviews were conducted in English, either face-to-face or online via Microsoft Teams, depending on participant preference and availability. A semi-structured interview guide was developed to explore providers’ experiences of PCC provision, perceived barriers and enablers, and contextual factors influencing practice. Interview duration ranged from 28 to 56 minutes. The shorter interviews reflected succinct, focused responses from providers that directly addressed the interview questions. All interviews were audio-recorded with participants’ permission.

The guide was informed by the 14 domains of the TDF (Cane *et al*. 2012) and drew on existing PCC research to ensure relevance to primary care and migrant health contexts (Mazza *et al*. 2013, Haith-Cooper *et al*. 2018, Sissons *et al.* 2020). Open-ended questions and prompts were used to allow flexibility and enable participants to raise issues beyond the predefined framework, consistent with recommendations for theory-informed qualitative research (McGowan *et al*. 2020). Example questions are: ‘What does preconception care mean to you in your practice?’, ‘How do you practise preconception care with African migrant women?’, ‘Who do you think should be responsible for providing preconception care in primary care?’, ‘What challenges have you faced in doing so?’, and ‘What would help improve preconception care provision for African migrant women in Victoria?’ The interview guide is provided as Supplementary material (Supplementary File S1). Interviews were transcribed verbatim, de-identified, and imported into NVivo version 15 for data management and to support analysis.

### Data analysis

Semi-structured interviews were analysed using a hybrid deductive–inductive thematic approach (Chew *et al*. 2026). Deductive coding was guided by the TDF (Michie *et al*. 2011, Cane *et al*. 2012) to ensure coverage of established determinants of behaviour, while inductive coding allowed new concepts to emerge directly from the data. Analysis began with data familiarization, during which transcripts were read repeatedly to achieve immersion and develop an initial understanding of participants’ accounts. Transcripts were then coded line-by-line, with initial codes assigned to discrete segments of text representing experiences, practices, or perceptions related to PCC provision. Codes were mapped to relevant TDF domains. Within and across these domains, an inductive process was then used to refine the coded data within domains, identify patterns in meaning, and develop higher-order subthemes grounded in participants’ views. Consistent with guidance for theory-informed qualitative analysis, the TDF was applied flexibly as an organizing framework rather than a prescriptive reporting structure, allowing participants’ perspectives to guide theme development (Atkins *et al*. 2017, McGowan *et al*. 2020). Following domain-level synthesis, subthemes were mapped to the COM-B model to identify the behavioural drivers influencing PCC provision. Regular discussions with the research team supported credibility and confirmability. For example, accounts describing limited consultation time, busy clinics, and difficulty extending PCC discussions were coded as time and workflow constraints. These data were mapped to the TDF domain ‘Environmental context and resources’ and interpreted as ‘Opportunity’ within the COM-B, because they reflected service conditions that shaped whether providers could initiate or continue PCC discussions.

### Ethical considerations

Ethical approval for this study was obtained from the Deakin University Human Research Ethics Committee (reference number 2024-021). All participants received a plain language statement outlining the study purpose, procedures, the voluntary nature of participation, and confidentiality measures. Written or electronic informed consent was obtained prior to participation. Participant anonymity was preserved using generic identifiers (e.g. PCP1–PCP11), and all data were stored securely in accordance with institutional data governance requirements. African community members and organizations were not formally involved in the study design, conduct, or analysis of the data.

## Results

### Participant characteristics

Participants ranged in age from 27 to 56 years, and more than half (54%) had more than 5 years’ experience working in primary care. The sample included seven female and four male providers (Table 1).

**Table 1:** Participant characteristics (*n* = 11).

Characteristic	Value
Age (years), mean (range)	40.6 (27–56)
Gender, *n* (%)	
Female	7 (63.6)
Male	4 (36.4)
Profession, *n* (%)	
General practitioner	4 (36.4)
MCH nurse	3 (27.2)
Practice/refugee nurse/nurse practitioner	4 (36.4)
Years of experience, *n* (%)	
1–5 years	5 (45.5)
5+ years	6 (54.5)

Findings are organized using the COM-B model to describe factors shaping the provision of PCC, with relevant TDF domains specified within each section. Across the data, PCC appeared to be delivered opportunistically as preventive care rather than as part of the standard care, with discussions shaped by appointment length, interpreter access, continuity, and trust. Providers’ accounts identified influences related to Capability (knowledge, skills, confidence, and staged delivery of PCC), Opportunity (time, funding, resources, practice systems, service contexts, language access, continuity, and social influences), and Motivation (perceived benefits of PCC, professional roles and responsibilities, and the need to increase PCC visibility in routine care and community settings).

### Capability to provide PCC

Providers described capability in relation to how they understood PCC, the knowledge sources they drew on, the gaps in training they experienced, and the clinical and communication skills they used to initiate, sequence, and deliver PCC discussions in practice. Capability involved more than knowing PCC components; providers also needed confidence to recognize PCC as structured preventive care and to raise pregnancy preparation appropriately.

#### Understanding of PCC and its scope

Providers’ descriptions of PCC reflected varied understandings of its scope and purpose (TDF domain: Knowledge). Most participants were familiar with individual PCC components, particularly biomedical and lifestyle-related elements, but fewer described PCC as a clearly defined or structured package of care delivered prior to pregnancy. As one GP stated, ‘*preconception consultation is like any consultation*’ (PCP3), while one practice nurse framed PCC as ‘*just all the check-ups*’ tailored to individual risk (PCP5).

Providers commonly described addressing nutritional supplementation, iron status, vaccination, screening, and chronic disease management as part of PCC-related care. A practice nurse explained that preconception discussions often focused on physiological readiness, stating that ‘*the blood level of iron and multivitamins in preconception has to be at least within the normal range*’ (PCP1). Vaccination timing was also highlighted, particularly for live vaccines, with a GP noting, ‘*we advise not to get pregnant for at least 6 weeks after the last dose of rubella or varicella vaccination*’ (PCP8). The same GP added, ‘*we routinely discuss folic acid, iron, vitamin D, calcium if needed, and immunization*’ (PCP8).

#### Sources of PCC knowledge

Clinical guidelines or institutional resources were explicitly referenced by some participants when describing PCC practice (TDF domain: Knowledge). These included professional guidelines and hospital-based information sources. As one GP explained, ‘*the RACGP Red Book specifies behavioural interventions and prevention and promotion of health for women planning pregnancy*’ (PCP7). An MCH nurse noted, ‘*we use the Royal Women’s Hospital website and 1800 My Options quite a lot*’ (PCP11).

#### Knowledge gaps and training needs

Alongside accounts of broad familiarity with PCC components, some providers reported limited expertise and minimal formal training in PCC (TDF domains: Knowledge and Skills). Training gaps were not limited to general PCC awareness; they also influenced providers’ confidence in addressing more complex preconception issues, such as medication safety and mental health conditions. One practice nurse stated: ‘*I don’t have much expertise… I know the common ones about vaccination, genetic screening, blood tests… but I’m not knowledgeable*’ (PCP2), and noted, ‘*I never took training on PCC… it is important to include it as one training package in general practice*’ (PCP2). Providers linked improved PCC knowledge with greater confidence and more consistent advice, with one GP stating, ‘*It is only with good knowledge that practitioners can feel confident … continuous awareness-raising and training are important*’ (PCP7).

Providers described limited awareness of PCC among African migrant and refugee women, particularly newly arrived women, as a knowledge-related barrier that increased the need for provider education during consultations. A nurse practitioner explained that ‘*newcomers are disadvantaged because of lack of information … they don’t know this can be preventable*’ (PCP5). An MCH nurse similarly noted that the barrier was ‘*not just accessibility, but not knowing the services exist’* (PCP6).

#### Skills and confidence in initiating PCC

Providers described drawing on a range of clinical and communication skills when delivering PCC-related care (TDF domain: Skills). The clinical skills identified included general health assessments, investigation ordering, and medication review. One GP described a typical PCC-related assessment as starting with ‘*a general check-up, including weight, height, blood pressure, eating habits, and lifestyle*,’ followed by basic investigations (PCP4). Medication review was frequently emphasized, particularly for women using medications that could harm a developing baby if taken during pregnancy. As another GP explained, ‘*if you are epileptic, those medications may have a risk on the unborn child, so we need to change this medication*’ (PCP3).

Skills for introducing and delivering PCC were described as particularly important when discussions arose opportunistically during routine consultations. One GP explained using cues such as contraception refills to initiate discussions, for example, ‘*if it’s a refill visit, I ask about side effects, duration, and pregnancy plans*’ (PCP7). A refugee health nurse similarly described adjusting their language to reduce discomfort and avoid assumptions; for example, asking, ‘*do you see yourself in the next few years having more children?*’ (PCP6).

Confidence in initiating and delivering PCC varied across participants (TDF domain: Beliefs about capabilities). For some, PCC was embedded in routine practice, while others reported limited experience. One practice nurse stated plainly, ‘*I’ve never done it in my career*’ (PCP2). Perceived confidence was also tied to professional development needs, with an MCH nurse noting that ‘*professional development is needed … because advice could really vary*’ (PCP9). In contrast, a refugee health nurse expressed confidence, stating, ‘*yeah, I think I am skilled, in all honestly*’ (PCP1).

#### Staged delivery and timing of PCC discussions

Preconception care was described as being delivered opportunistically and in a staged manner across multiple encounters rather than a single discussion (TDF domain: Behavioural regulation). This staged approach was particularly evident in maternal and child health settings, where earlier visits focused on contraception and later visits introduced preparation for future pregnancies. As one MCH nurse explained, ‘*earlier on after the first child, I discuss contraception, but when they come back 18 months later, then I talk about preparing for future pregnancies*’ (PCP11). The same participant also described revisiting PCC repeatedly over time: ‘*I think you need to… touch on this conversation and build on it at each visit… for some… one or two conversations… for some… four or five conversations*’ (PCP11).

Many providers reported that PCC conversations were typically provider-initiated, with timing influenced by when women might be considering another pregnancy. One GP noted, ‘*patients usually come to the clinic for reasons other than PCC, but I take the opportunity to address PCC during those visits*’. Similarly, another GP described using contraception-related visits as cues to introduce PCC and arrange follow-up for more detailed discussion: ‘*I found it more relaxing to discuss pregnancy plans when I am already in that discussion of contraceptive options … one can come for implant removal, then I ask what are your plans?*’ (PCP7). An MCH nurse also noted that ‘*it usually wouldn’t come from the woman. We would initiate conversations about their health*’ (PCP10) and suggested that ‘*18-month or two-year checks*’ were a time when women ‘*start to think about having another child*’. Participants also described integrating lifestyle risk and medication-related advice into staged PCC discussions. This included advice about weight management, exercise, supplementation, and medication safety before pregnancy. One GP explained that ‘*if there are weight issues, we recommend starting exercise now*’ (PCP4), while another GP emphasized the need to review and adjust medicines before pregnancy: ‘*We warn them… medication may affect the unborn child. We need to consult or change medication before pregnancy*’ (PCP3).

### Opportunity to provide PCC

Many providers described opportunity as shaped less by willingness to provide PCC and more by whether PCC was practically and socially supported within the primary care context. Key conditions included short appointments, funding and billing arrangements, resources for consistent messaging, continuity and trusted relationships, interpreter access, and whether PCC could be shared across the care team (TDF domains: Environmental context and resources, and Social influences).

#### Time, workload, and funding

In general practice settings, short appointments, high patient volumes, and competing clinical priorities constrained opportunities for PCC (TDF domain: Environmental context and resources). Providers noted that PCC could sometimes be introduced briefly, but comprehensive counselling and preventive planning often required longer or multiple consultations. One GP described the cumulative effect of time pressure: ‘*The main challenge in general practice is it's always busy. You’ve got 10 minutes for a person… you’re running late for the next patient*’ (PCP7). In these accounts, PCC was valued but difficult to operationalize within standard consultations; providers often raised it briefly after addressing the presenting concern. Funding structures were also positioned as limiting the opportunity to provide essential PCC (TDF domain: Environmental context and resources). A nurse practitioner noted that ‘*the timing and the item number allocated… doesn’t match*’ (PCP5), describing a gap between the time required for PCC and what routine primary care funding accommodates. Another provider emphasized system-level trade-offs: ‘*The more time we need for appointments, the fewer families we can see*’ (PCP10). Some providers suggested that task sharing could partly address these constraints by allowing nurses, nurse practitioners, and midwives to provide components of PCC that required more time, particularly education and initial assessment. As one GP explained, ‘*nurse practitioners can book them… most of it can be done by nurses… and midwives*’ (PCP7).

#### Resources and practice systems

Providers described clinic resources and practice systems as ways to increase PCC visibility and reinforce advice during time-limited consultations (TDF domain: Environmental context and resources). These included brochures, posters, waiting-room information, and clinic websites. One GP suggested that clinics could list ‘*preconception… and pregnancy care*’ on their websites… (PCP3). Providers also described using practice software to support PCC education and reinforce verbal advice, with one GP explaining, ‘*there’s patient education resources on the software, so I print and give them that*’ (PCP4).

#### Service contexts and language access

Providers highlighted that opportunities for PCC varied by service context, particularly when early and repeated contact enabled PCC to be introduced across routine care encounters (TDF domains: Environmental context and resources, and Social influences). Some providers identified maternal and child health services as settings where PCC discussions could occur opportunistically. An MCH nurse described these encounters as offering ‘*plenty of little windows to chat about that*’ (PCP9). Refugee health nurses were described as having early contact with newly arrived clients and, therefore, having a unique opportunity to prompt discussions on reproductive planning to help women navigate PCC support in general practice: ‘*we see them before they ever enter the general health system*’ (PCP6).

Interpreter access was described as essential for meaningful PCC discussions, particularly given the complexity and sensitivity of the topics involved (TDF domains: Environmental context and resources, and Social influences). Some providers reported that without interpreters, they were unlikely to discuss PCC in depth. One practice nurse noted that ‘*if you don’t have an interpreter, you’re not going to go into something like PCC properly*’ (PCP2). However, providers also indicated that use of an interpreter did not automatically make PCC discussions acceptable or comfortable for women. Trust, familiarity, and privacy supported or constrained communication, particularly when interpreters were perceived as connected to women’s communities (TDF domain: Social influences). An MCH nurse reported that some women ‘*can be resistant to using interpreters because they may know the interpreters… part of their community*’ (PCP10).

Providers also described women’s engagement with PCC as shaped by structural and material circumstances, including education, health literacy, migration pathways, displacement, settlement experiences, and socioeconomic conditions (TDF domain: Environmental context and resources). As a refugee nurse explained, ‘*… if they feel listened to, respected, and clearly communicated with, then they usually don’t mind which GP they see*’ (PCP6). A GP described variation among African migrant women according to ‘*literacy rates, age group… people who have gone through displacement*’ (PCP7). Another GP said, ‘*Most people with higher socioeconomic status and education level do everything with consultation, do check-up every year’* (PCP8).

#### Teamwork and continuity of care

Providers described PCC as more feasible when care was organized to support teamwork and continuity, particularly when nurses or other team members could spend more time on education and follow-up (TDF domains: Social influences, and Environmental context and resources). Some participants suggested that PCC activities could be shared across the primary care team, with one GP noting that ‘*Most of it can be done by nurses in relation to education… if you could have a nurse present, the nurse might have more time to explore those kinds of areas*’ (PCP7). A nurse practitioner emphasized team-based approaches, suggesting ‘*It should be a multidisciplinary team because not only RNs can do it… it must be GPs, nurse practitioners, and RNs, we all have to work together*’ (PCP5). One MCH nurse also noted that PCC discussions were easier when a trusted relationship had been established over time, with one explaining that ‘*whoever's offering the continuity of care is the best person for the conversation because they've had the opportunity to build rapport and gain trust*’ (PCP11).

#### Social influences shaping engagement with PCC

Providers described social and cultural influences shaping women’s engagement with PCC, including trust, familiarity, and broader social circumstances (TDF domain: Social influences). These influences interacted with service access, communication quality, and the respect with which PCC was introduced. Some reported that PCC was not commonly prioritized and, for some women, was viewed as unfamiliar or unnecessary. A practice nurse described PCC as something some women saw as ‘*a ‘white people’s thing’*’ (PCP1). Providers further referred to religious and cultural beliefs as shaping attitudes towards contraception, screening, and pregnancy. For example, a nurse practitioner noted that ‘*among some groups, pregnancy was accepted as a blessing and contraception was not usually considered*’ (PCP5). Providers also suggested that women were more open when they felt understood by someone from a similar cultural background. One practice nurse stated, ‘*As an African woman myself, I find it easier for women to talk openly with me… it was easier for the patients to listen to their kind*’ (PCP1).

### Motivation to provide PCC

Providers described motivation to provide PCC in relation to the importance they attached to PCC, the value they placed on increasing its visibility, and their professional responsibility for providing education and care (TDF domains: Beliefs about consequences, Goals, and Social/professional role and identity). Although providers valued PCC, motivation alone did not translate into routine provision without clear guidance, consultation time, interpreter support, and team-based pathways. Providers linked PCC to prevention and improved outcomes for women and babies (TDF domains: Beliefs about consequences). A practice nurse described PCC as ‘*… crucial to have a healthy population, to decrease burden on the public and the healthcare system*’ (PCP2), while an MCH nurse explicitly connected maternal health to child outcomes: ‘*Well it’s both … maternal health and … child’s development*’ (PCP10).

Providers also emphasized the need for PCC to be more visible in routine care and community settings, reflecting both a motivational priority and the environmental conditions required to support delivery (TDF domains: Goals and Environmental context and resources). A practice nurse stated, ‘*I really believe that at every doctor visit, patients should have access to education… also in schools so young girls… are aware*’ (PCP1). The same provider also described community-based education as essential: ‘*There are multiple multicultural communities that each council is aware of… to deliver education sessions for each of these communities…*’ (PCP1).

#### Professional role, duty of care, and education as core work

Providers described motivation to provide PCC in relation to professional role, scope of practice, duty of care, and education (TDF domain: Social/professional role and identity). Some viewed PCC as a routine part of professional responsibility. One MCH nurse stated, ‘*Provision of pre-pregnancy care is part of our practice, and we accept it as one of our professional scopes of practice*’ (PCP11). Providers also linked this role to safeguarding the health of women and babies. One practice nurse stated, ‘*Any safety that comes to the patient is part of my role*’ (PCP1). Education was also described as central to this professional role. *One GP stated, ‘So patient education is the core’ (PCP8),* while a nurse practitioner stated, ‘*Part of my role, whether she takes it or not, I give them education*’ (PCP5).

## Discussion

We explored primary care providers’ perspectives on barriers to and enablers of PCC provision for African migrant and refugee women in Victoria, Australia. In COM-B terms, the inconsistent PCC delivery was shaped largely by constraints in providers’ capability and opportunity, including variable PCC knowledge, confidence, time, interpreter access, resources, and role clarity, while motivation to provide PCC was generally strong. These findings align with evidence that PCC remains unevenly implemented in primary care despite guideline endorsement (Mazza *et al*. 2013), and extend the evidence base by highlighting the equity dimensions of PCC delivery for refugee and migrant women.

Providers’ accounts indicated that PCC was rarely conceptualized as a distinct, structured package of care. Instead, PCC was commonly understood and delivered as a set of biomedical activities embedded in routine encounters such as supplementation, vaccination, and medication review. This pattern is consistent with the literature showing that primary care providers often focus on discrete risk factors rather than comprehensive preconception planning (Mazza *et al*. 2013, Caut *et al*. 2025). The lack of a shared and standardized understanding of PCC in the literature may help explain the variation in how, when, and for whom PCC is delivered across providers and settings, with care often occurring opportunistically rather than through a structured model (Shawe *et al*. 2015, Caut *et al*. 2025). From a health promotion perspective, such fragmentation limits the capacity of PCC to function as an upstream preventive strategy, including its potential to address biomedical, behavioural, and social determinants of maternal and child health before pregnancy (WHO 2013, Bateson and Black 2018).

Providers identified limited formal training and uncertainty about PCC scope as influencing their confidence in initiating PCC discussions. Although many providers demonstrated practical knowledge of PCC components, uncertainty about the full scope of PCC and limited exposure to structured training were evident. This finding is consistent with broader primary care literature indicating that limited access to training and professional development can undermine clinicians’ confidence in delivering preventive care (Goossens *et al*. 2018a, Hammarberg and Taylor 2019, Caut *et al*. 2025). Migrant and refugee women’s engagement with preventive and reproductive health care is shaped by migration-related disadvantage and health system factors, including barriers to navigation, communication, and access (Mengesha *et al*. 2017, Lebano *et al*. 2020, Billett *et al*. 2022). In this context, PCC approaches that rely on women requesting care or already being aware of PCC may be less likely to reach those who are least well-positioned to navigate and access preventive services (Mengesha *et al*. 2017, Lebano *et al*. 2020, Dorney *et al*. 2025).

Providers described PCC delivery as largely opportunistic, commonly introduced during consultations focused on contraception, medication review, chronic disease management, or postnatal care, and often staged across multiple encounters. Opportunistic PCC reflects broader challenges in integrating prevention into time-limited primary care workflows and has been widely described across settings (van Voorst *et al*. 2016, M'Hamdi *et al*. 2017, Poels *et al*. 2017), including in Australian primary care (Walker *et al*. 2021). While staged PCC discussions may enable clinicians to prioritize issues across limited consultations, this approach depends on continuity and reliable follow-up systems. This is equity-relevant because migrant and refugee women often face barriers to continuity of care, including limited knowledge of the health system, difficulties navigating complex service pathways, and structural constraints on access. These factors collectively diminish their likelihood of completing staged preventive care (M'Hamdi *et al*. 2017, Mengesha *et al*. 2017, Lebano *et al*. 2020).

Time, workload, and funding constraints were prominent in providers’ accounts and help explain why PCC was deprioritized when consultations were dominated by acute or competing concerns. Similar barriers are consistently reported in provider-focused PCC research, including limited consultation time, competing priorities, and insufficient structural supports for preventive care (Mazza *et al*. 2013, M'Hamdi *et al*. 2017, Goossens *et al*. 2018b, Caut *et al*. 2025). Evidence syntheses further suggest that improvements in preconception and antenatal preventive care are more likely when implementation strategies address workflow and system supports, rather than relying on clinician intention alone (Doherty *et al*. 2022, Withanage *et al*. 2023).

Providers characterized interpreter access as fundamental to meaningful PCC counselling, highlighting limitations that arose when interpreters were unavailable or when women were reluctant to use interpreters from their own communities. Viewed through Levesque *et al*.’s access framework, these findings suggest that PCC access was shaped not only by service availability, but also by the approachability, acceptability, and appropriateness of care for African migrant and refugee women (Levesque *et al*. 2013). These concerns are consistent with Australian evidence that language barriers and inadequate interpreter access can compromise communication quality, patient safety and equity in care for migrant and refugee communities (Victorian Government 2018, Billett *et al*. 2022, FECCA 2022). Providers also emphasized trust and communication as central to engaging women in sensitive discussions about pregnancy intentions and reproductive health. These findings align with broader work showing that culturally responsive, respectful communication shapes access, engagement and acceptability of care for migrant and refugee women (Mengesha *et al*. 2017, Lebano *et al*. 2020, Pentecost and Meloni 2020, Rogers *et al*. 2020). PCC commonly involves nuanced counselling (including medication safety, supplementation, vaccination and optimization of chronic conditions), and inconsistent language access and variable cultural responsiveness risk limiting informed choice and narrowing PCC to minimal or biomedical-only messages (WHO 2013, RACGP 2025).

Professional roles and team-based models were described as potentially enabling PCC delivery but also marked by ambiguity around responsibility and ownership. While some participants viewed PCC as core work, others were unclear about whether it fell within their role, indicating that when responsibility is not clearly defined, PCC is more likely to depend on individual provider initiative than to be delivered consistently across roles and settings. The potential role of nurses in PCC aligns with team-based primary care and task-sharing approaches that expand preventive care capacity (Goossens *et al*. 2018b, Walker *et al*. 2021). However, task-sharing should not be interpreted as simply shifting PCC responsibilities from GPs to nurses, particularly when nurses also face time, staffing, and funding constraints. Evidence indicates that team-based PCC can remain underutilized when roles are unclear and organizational supports such as training, protocols, and resources are limited (Goossens *et al*. 2018b, Walker *et al*. 2021, Caut *et al*. 2025). Strengthening shared pathways and practice systems to support consistent care delivery is also consistent with implementation evidence that multi-component strategies, such as combining education with prompts, templates, and workflow supports, can improve preventive care delivery (Doherty *et al*. 2022).

Continuity of care was commonly described as facilitating PCC through trust and repeated engagement; however, reliance on continuity alone may disadvantage women who experience fragmented access due to structural and social constraints. Such limitations are well documented in migrant and refugee health literature, including barriers related to service navigation, language and communication, and gaps in culturally-responsive care that can disrupt ongoing engagement (Mengesha *et al*. 2017, Lebano *et al*. 2020, Rogers *et al*. 2020, Olcon *et al*. 2023).

Providers’ accounts reflected a strong commitment to providing PCC, suggesting that lack of motivation is unlikely to be the primary barrier in this setting. This interpretation is consistent with the COM-B framework, which posits that although motivation is necessary, it is insufficient in directing behaviour change when capability and opportunity are constrained (Michie *et al*. 2011). Providers also described tailoring PCC based on women’s perceived readiness and contextual circumstances. While responsiveness to women’s priorities may support culturally safe practice, reliance on subjective assessments can contribute to inequalities in delivery, particularly when trust, interpreter-mediated communication, and comfort in discussing sensitive topics influence disclosure and engagement in care (Mengesha *et al*. 2017, Lebano *et al*. 2020, Pentecost and Meloni 2020, Rogers *et al*. 2020).

Taken together, the findings suggest that strengthening PCC equity for African migrant and refugee women will require attention to how PCC is operationalized in routine primary care, including shared definitions of PCC scope, strengthened workforce capability, reliable access to interpreters, and practice systems that reduce reliance on opportunistic clinician discretion.

### Implications for health promotion and primary care practice

Consistent with the COM-B model, providers’ accounts point to actionable targets for strengthening PCC provision in primary care. From a health promotion perspective, this requires reorienting primary care so that PCC is offered proactively, rather than relying on women’s requests or individual providers’ initiative. These targets include training and guidance to strengthen capability; workflow supports, reliable interpreter access, team-based pathways, and adequate consultation structures to strengthen opportunity; and positioning PCC as core preventive work to support motivation (Michie *et al*. 2011). Future research should evaluate the acceptability, feasibility, and equity impacts of these targets in primary care.

Equity-oriented PCC also requires culturally responsive delivery conditions. Australian evidence highlights persistent system-level barriers for culturally and linguistically diverse communities, including inadequate interpreter access and limited cultural responsiveness, which can restrict access to equitable preventive care (Victorian Government 2018, Billett *et al*. 2022, FECCA 2022).

### Strengths and limitations

A key strength of this study is its focus on primary care providers’ perspectives on PCC for African migrant and refugee women, a population that is under-represented in the preconception and health promotion literature. By including practice or refugee health nurses and MCH nurses, the study provides a detailed understanding of PCC delivery conditions and role perceptions across different primary care settings. Participants were those with experience of providing care to African migrant and refugee women, and most had African backgrounds, strengthening the study through perspectives grounded in clinical practice with culturally and linguistically diverse populations. The use of theory-informed qualitative methods supported systematic exploration of behavioural, social, and contextual factors shaping PCC provision, while still allowing participants’ experiences to guide the analysis.

The study also has limitations. As a qualitative study conducted in Victoria, the findings are context-specific and may not be directly transferable to other health systems or settings with different models of primary care, funding arrangements, or migrant populations. Participants were recruited through purposive and snowball sampling, which may have resulted in the inclusion of providers with greater interest or engagement in women’s health or migrant care. Interview length varied based on provider availability and response style, but all interviews addressed the core questions on barriers and enablers to PCC provision.

## Conclusion

This study highlights the complex, interconnected factors that shape the provision of PCC for African migrant and refugee women in primary care. While providers recognized the importance of PCC and demonstrated commitment to preventive practice, delivery was shaped by variation in knowledge and confidence, time and resource constraints, and uncertainty regarding professional roles. Therefore, strengthening equitable PCC provision requires reorienting primary care in practical ways: consultation structures that allow sufficient time, reliable interpreter access, clear role expectations, and routine opportunities to raise PCC proactively during contraception, postnatal, medication review, chronic disease, and general health visits.

## Supplementary Material

daag091_Supplementary_Data

## Data Availability

The data underlying this article cannot be shared publicly due to the sensitive nature of the interview data and the risk of identifying individual participants. In accordance with the approved ethics protocol, and to protect participant privacy and confidentiality, the data are not available for sharing.
