# Supplementary material for: Primary care providers’ perspectives on barriers and enablers influencing preconception care provision for African migrant and refugee women: qualitative interviews
Source: Health Promot Int. 2026 Jul 17;41(4):daag091. doi: 10.1093/heapro/daag091 (PMC13394715; doi:10.1093/heapro/daag091)
Supplement: daag091_Supplementary_Data [file daag091_supplementary_data.docx]

**A semi-structured interview guide for primary care practitioners**

Demographic information: Years of experience, Professional role, Age, and Gender.

**Knowledge**

- What does pre-pregnancy care mean to you in your practice?
- What do you see as the main components of pre-pregnancy care?
- Are you aware of any national or international recommendations about care before pregnancy? Can you tell me what you know about them?

**Skills**

- How do you practice pre-pregnancy care with African migrant women?
- What components of care have you practised? - Provide any example

**Social/Professional Role and Identity**

- What are your opinions regarding providing pre-pregnancy care to women?
- Do you accept pregnancy care as part of your scope of practice?
- Who do you think should be responsible for providing pre-pregnancy care in primary care?

**Beliefs about capabilities**

- How feasible is it to offer pre-pregnancy care in your setting according to existing recommendations?
- What challenges have you faced in doing so?
- Are there specific problems in relation to providing pre-pregnancy care for African migrant women?
- Do you consider yourself well prepared to provide pre-pregnancy care in terms of knowledge and skills?

**Optimism**

- How optimistic are you that pre-pregnancy care can be provided effectively in your practice setting?
- What makes you feel hopeful or confident about providing pre-pregnancy care?
- Are there things that make you feel pessimistic or doubtful about providing pre-pregnancy care, especially for African migrant women?

**Beliefs about consequences**

- Do you believe that pre-pregnancy care has benefits? If yes, what are the benefits?
- What will happen if pre-pregnancy care is routinely practised in a primary care setting?

**Reinforcement**

- Are there any factors that encourage or reward you to provide pre-pregnancy care in routine practice?
- Are there any feedback mechanisms, incentives, or recognition that support pre-pregnancy care provision?
- Are there any factors that discourage you from providing pre-pregnancy care?

**Intentions**

- Do you intend to provide pre-pregnancy care routinely in your practice?
- How strong is your intention to raise pre-pregnancy care with women of reproductive age?
- What influences whether you decide to bring up pre-pregnancy care during consultations?

**Goals**

- How much of a priority is pre-pregnancy care in your day-to-day practice?
- What goals, if any, do you have in relation to providing pre-pregnancy care?
- How does pre-pregnancy care fit alongside your other clinical priorities?

**Memory, attention, and decision processes**

- What helps you remember to raise pre-pregnancy care in practice?
- Are there particular cues, situations, or types of visits that prompt you to discuss it?
- What makes it easier or harder to prioritise pre-pregnancy care during consultations?

**Environmental context and resources**

- How can work environment and resource issues influence your pre-pregnancy care practice?
- Do you have other priority activities that require more attention?
- How can they influence your pre-pregnancy care practice?

**Social influences**

- To what extent are people around you (colleagues and staff) supportive or a challenge in the provision of pre-pregnancy care?

**Emotion**

- Do you think that emotions could influence pre-pregnancy care provision?
  - How do you feel about providing preconception care to African women?

**Behavioural regulation**

- Are there conditions that can help primary care practitioners provide pre-pregnancy care?

**General reflection**

- What would help improve pre-pregnancy care provision for African migrant women in Victoria?
- Is there anything else you would like to share about providing pre-pregnancy care in this context?
